# Chemometric evaluation of *Saccharomyces cerevisiae* metabolic profiles using LC–MS

**DOI:** 10.1007/s11306-014-0689-z

**Published:** 2014-06-25

**Authors:** Mireia Farrés, Benjamí Piña, Romà Tauler

**Affiliations:** Institute of Environmental Assessment and Water Research (IDAEA), Spanish Council for Scientific Research (CSIC), Jordi Girona 18-26, 08034 Barcelona, Spain

**Keywords:** Metabolic profiling, Untargeted metabolomics, Metabolite identification, *Saccharomyces cerevisiae*, Multivariate curve resolution-alternating least squares, Partial least squares-discriminant analysis, Liquid chromatography–mass spectrometry

## Abstract

A new liquid chromatography mass spectrometry (LC–MS) metabolomics strategy coupled to chemometric evaluation, including variable and biomarker selection, has been assessed as a tool to discriminate between control and stressed *Saccharomyces cerevisiae* yeast samples. Metabolic changes occurring during yeast culture at different temperatures (30 and 42 °C) were analysed and the complex data generated in profiling experiments were evaluated by different chemometric multivariate approaches. Multivariate curve resolution alternating least squares (MCR-ALS) was applied to full spectral scan LC–MS preprocessed data multisets arranged in augmented column-wise data matrices. The results showed that sectioning the MS-chromatograms in different windows and analysing them by MCR-ALS enabled the proper resolution of very complex coeluted chromatographic peaks. The investigation of possible relationships between MCR-ALS resolved chromatographic peak areas and culture temperature was then investigated by partial least squares discriminant analysis (PLS-DA). Selection of most relevant resolved chromatographic peaks associated to yeast culture temperature changes was achieved according to PLS-DA-Variable Importance in Projection scores. A metabolite identification workflow was developed utilizing MCR-ALS resolved pure MS spectra and high-resolution accurate mass measurements to confirm assigned structures based on entries in metabolite databases. A total of 65 metabolites were identified. A preliminary interpretation of these results indicates that the strategy described in this study can be proposed as a general tool to facilitate biomarker identification and modelling in similar untargeted metabolomic studies.

## Introduction

Cell metabolites describe the physical and chemical characteristics of organisms. Metabolomics aims to measure the global, dynamic metabolic response of living complex multicellular systems to biological stimuli or genetic manipulation (Nicholson and Lindon 2008). It determines changes in low molecular weight organic metabolites in complex biological samples. By identifying biochemical compounds whose concentrations have varied due to a biological stimulus, metabolomics allows uncovering new possible targets (biomarkers) for biochemical interpretation of biological changes.

Currently, a range of analytical platforms are used for metabolomic analysis, including direct infusion mass spectrometry (MS) (Højer-Pedersen et al. 2008), gas chromatography coupled to mass spectrometry (GC–MS) (Lu et al. 2008), two-dimensional GC coupled to MS (GC × GC–MS), liquid chromatography coupled to MS (LC–MS) (Bajad et al. 2006), capillary electrophoresis coupled to MS (CE–MS), and proton nuclear magnetic resonance (1H NMR) spectroscopy and Fourier transform infrared (FT-IR) spectroscopy. Complete chromatographic separation of the components of complex biological samples is often difficult to achieve. Despite the fundamental advantages of metabolomics, so far no metabolomic platform allows for the reliable complete separation, detection and identification of all metabolites. Actually, the analysis of the full metabolomes is a very difficult task due to the large chemical diversity of cellular metabolites (Villas-Bôas et al. 2005; Werf et al. 2007; Garcia et al. 2008; Theodoridis et al. 2012; Xu et al. 2014)

In general, targeted metabolomics approaches are directed to the detection and quantification of specific classes of compounds. In contrast, non-targeted metabolomics aims to study the widest possible range of compounds and enables the identification of most discriminatory metabolites that can be used as biomarkers (Glinski and Weckwerth 2006). A global non-targeted metabolomics in combination with multivariate data analysis aims to the isolation of previously unknown biomarkers specific to a particular biological stimulus.

LC–MS-based approaches are of particular importance for non-targeted metabolomics. Metabolites can be extracted with aqueous alcohol solutions and directly analysed. In principle, LC–MS does not require any prior pretreatment of samples to distinguish between different metabolite groups of interest and it is suitable for the detection of a wide range of metabolite classes. Depending on the type of chromatographic column used for the analysis, various metabolite groups can be reliably analyzed using LC–MS. High mass spectrometry resolution with electrospray ionization (ESI) is the preferred method in terms of universality, high throughput, resolution and sensitivity (Niessen 1999).

When metabolomic profiles are analysed by LC–MS in full spectral scan mode, some drawbacks, like baseline distortion, retention time peak shifting and possible peak shape distortions from one chromatographic run to another, and possible strong peak coelution problems can appear. Different chemometric methods can be used to reduce the effects of these drawbacks, such as baseline correction methods (Eilers 2004), peak alignment methods (Savorani et al. 2010), warping methods (Nielsen et al. 1998), wavelets methods (Walczak et al. 1996) and multivariate curve resolution (Parastar and Akvan 2014). In particular, because of the ubiquitous existence of the large number of overlapping embedded peaks, multivariate curve resolution methods can be very useful and necessary to achieve the goals of metabolomics studies by full spectral scan LC–MS.

*Saccharomyces cerevisiae* is a budding yeast species, which comprises a group of unicellular fungi belonging to *Ascomycetes* phylum. *S.* *cerevisiae* has been used as a model for higher eukaryote species in biology because its similar metabolism (Sherman et al. 2002; Castrillo and Oliver, 2006). In this study the LC–MS metabolomics approach is coupled to different chemometric methods, such as MCR-ALS and PLS-DA, to explore the changes observed in the metabolite profiles of *S.* *cerevisiae* when it is cultivated at different temperatures. In this work, a new strategy using Multivariate Curve Resolution-Alternating Least Squares (MCR-ALS) (Tauler 1995; Peré-Trepat et al. 2005) is proposed as a general approach for proper investigation and resolution of complex and extensive LC–MS data sets (in full spectral scan mode), where huge amounts of information can be uncovered, including strongly hidden coeluted and embedded unknown chromatographic peaks. Related approaches have been already proposed in previous works (Pérez et al. 2009; Szymańska et al. 2009; Siano et al. 2011) to solve similar coelution problems in metabolomics, but this work goes a step further and apart from their resolution, metabolites are also identified by their exact mass. In addition, Partial Least Squares-Discriminant Analysis (PLS-DA) (Barker and Rayens 2003) is applied to the MCR-ALS results to investigate what metabolites were more influenced by the temperatures changes on yeast cultures, acting therefore as a possible biomarkers of temperature stimulus on yeast cultures.

## Experimental

### Chemicals

Pure metabolites threonine, valine, isoleucine, glutamic acid, adenosine monophosphate (AMP), adenosine triphosphate (ATP), 3-phosphoglyceric acid, glucose-1-phosphate, fructose-6-phosphate, fructose-1,6-biphosphate, itaconic acid, succinic acid and citric acid were obtained from Sigma-Aldrich (St. Louis, USA). Stock individual standard solutions (500 μg mL^−1^) were prepared dissolving accurate amounts of pure standards in acetonitrile:water 1:1. Two standard mixture samples of these compounds were prepared at 10 and 20 μg mL^−1^ concentration levels in acentonitrile:water 1:1. Ethanol, Acetonitrile and HPLC grade water were obtained from Merck (Darmstadt, Germany).

### Culture conditions

Yeast strains W303a were grown in glass cultured tubes overnight at 30 °C and 150 rpm in non-selective medium (yeast extract peptone dextrose, YPD, 5 g L^−1^ yeast extract, 10 g L^−1^ peptone, 20 g L^−1^ glucose). Eight shake flask cultures were performed in 100-mL flasks with 50 mL medium. Samples culture media were inoculated with 50 μL of yeast pre-cultures in YPD medium and incubated for at 30 °C and 150 rpm. After 7 h four flask cultures were incubated at 30 °C and the other four flask cultivations at 42 °C, in both cases for 1 h.

### Quenching and extraction of metabolites

Four types of samples were investigated (i) one standard mixture at 20 μg mL^−1^ (ii) one standard mixture at 40 μg mL^−1^; (iii) four yeast samples cultivated at 30 °C; and (iv) four yeast samples cultivated at 42 °C. The same analytical pretreatment was applied in biological and standards samples. Metabolites extraction procedure was performed using three blank (without yeast) samples.

After culture, samples were poured to a 50 mL Falcon tubes and the metabolism of the cultures samples was rapidly inactivated cooling down the mixture on ice. Once cooled down, all tubes were centrifuged at 4,000 rpm for 15 min at 4 °C. The supernatant was removed and yeast pellets remained into the Falcon tube. Pellet was then cleaned up with phosphate buffered saline (PBS). At this point, the two standard mixture samples at 20 and 40 μg mL^−1^ were added. A volume of 25 mL of PBS was poured into each sample to adjust their pH to 7.4. Falcon tubes were centrifuged at 4,000 rpm for 10 min at 4 °C. Again the supernatant was removed. This step was repeated twice. All through the clean-up procedure, samples were kept in cold.

Extraction of yeast metabolites was carried out according to the procedure previously described (Gonzalez et al. 1997). Metabolites extraction was performed into 50 mL Falcon tubes, adding 5 mL of solvent (75 % ethanol) to the cell pellet and further incubation of the suspension for 3 min at 80 °C. After cooling down the mixture on ice, sample volume was concentrated and dried by evaporation using nitrogen gas. The residue was resuspended to a final volume of 0.5 mL with the LC mobile phase (95 % acetonitrile). Prior to pouring the final volume to a vial, it was filtered through 0.2 μm GHP membranes (GHP, Acrodisc Syringe Filters, Pall Life Sciences, USA) to further ensure removal of any residual protein/debris before LC analysis.

### LC–MS

An Accela liquid chromatograph (Thermo Scientific, Hemel Hempstead, UK) equipped with a quaternary pump, a thermostated autosampler and a TSKgel Amide-80 5-μm (100 × 2.0 mm) column purchased from Tosoh (Tokyo, Japan) was used. LC solvents were 0.5 mM ammonium acetate in 90 % acetonitrile at pH 5.5 (solvent A) and 2.5 mM ammonium acetate in 90 % acetonitrile in 60 % acetonitrile at pH 5.5 (solvent B). The gradient elution was as follows: *t* = 0, 5 % B; *t* = 8, 60 % B; *t* = 12, 95 % B; *t* = 17.5, 95 % B; *t* = 20, 5 % B; *t* = 30, 5 % B. Injection volume was 5 μL and flow rate was 0.3 mL/min.

An LTQ Orbitrap Velos mass spectrometer (Thermo Scientific, Hemel Hampstead, UK) equipped with an ESI source in positive mode was used to acquire mass spectra profiles in full scan mode. Operation parameters were: source voltatge, 3.5 kV; sheath gas, 40 (arbitrary units); auxiliary gas, 10 (arbitrary units), sweep gas, 5 (arbitrary units); and capillary temperature, 275 °C. The acquired mass range was from 50 to 1000 Da. The mass spectrometer was interfaced to a computer workstation running Xcalibur 2.1 software for data acquisition and processing.

### Data import

Full scan MS spectra of different chromatographic runs were saved in raw mode in Xcalibur software 2.0 (Thermo Xcientific, San Jose, CA) and converted to mzXML by ReAdW software (Seattle Proteome Center 2014) and imported to MATLAB (The Mathworks Inc. Natick, MA, USA) computer environment with the mzxmlread.m function from the Bioinformatiocs Toolbox 3.0.

## Chemometric data preprocessing and analyses

Chemometric data analysis included different multivariate data analysis methods like Principal Component Analysis (PCA), Partial Least Squares-Discriminant Analysis (PLS-DA) and Multivariate Curve Resolution-Alternating Least Squares (MCR-ALS) (Jaumot et al. 2005). Matlab R2007a (Mathworks Inc. Natick, MA, USA) and PLS Toolbox 5.8.1 (Eigenvector Research Inc., Wenatchee, WA, USA) were used as computer programming environments for all chemometric analyses.

### Data preprocessing

Each analysed yeast sample produced a raw full scan MS chromatogram which was imported to Matlab and initially binned to their integer mass to facilitate faster processing and chemometric analysis. Each full scan MS chromatogram was stored in a data matrix with dimensions of 3,587 rows (retention times, ranging from 0 to 30 min) and 951 columns (mz intensity values, ranging from 50 to 1,000 Da).

Raw full scan MS chromatograms were size reduced, giving a total number of 546 *mz* values within the mass range between 55 and 600 Da. MS chromatograms were then interpolated to the same retention times giving a total number of 2020 retention times, ranging from 0 to 17 min. Therefore the final size of every data matrix corresponding to a full scan MS chromatogram of a yeast sample was of 2020 rows by 546 columns. Baseline and background contributions were corrected by subtraction of the mean chromatogram of the blank samples. To have most of data values at reasonable units (between 0 and 2), the intensity scale of all chromatograms was divided by 10^8^. The resulting preprocessed data matrices from all yeast samples were then analysed by MCR-ALS.

On the other hand, the eight Total Ion Current (TIC) yeast MS chromatograms were also arranged altogether in a single TIC data matrix (8 rows × 2,020 columns). Before its analysis, chromatographic peaks were appropriately aligned to compensate possible between run retention time shifts. The Correlation Optimized Warping (COW) method (Nielsen et al. 1998; Tomasi et al. 2004) was selected for this purpose. The application of this method required as input parameters, the segment *m,* which is the length of the sections in which the chromatogram is divided, the slack size *t,* which is the maximum chromatographic peak warping allowed and a reference chromatogram (Nielsen et al. 1998). These parameters were selected according to the method proposed in previous works (Skov et al. 2006). In order to improve the application of the peak alignment procedure, the TIC matrix (8 ×°2,020) was divided in two submatrices with dimensions of: 8 ×°1,300 and 8 ×°720, and COW alignment was then performed in each part individually. After alignment both windows were then rejoined. Before PCA, the already aligned TIC chromatograms in TIC data matrix were mean-centred.

### Data arrangement

Two types of data sets were analysed in this work: (i) the eight individual Total Ion Current (TIC) chromatograms arranged in a single TIC data matrix and (ii) the eight full scan MS chromatograms sectioned in different windows and arranged in different column-wise augmented data matrices (see below).

**Fig. 1:**
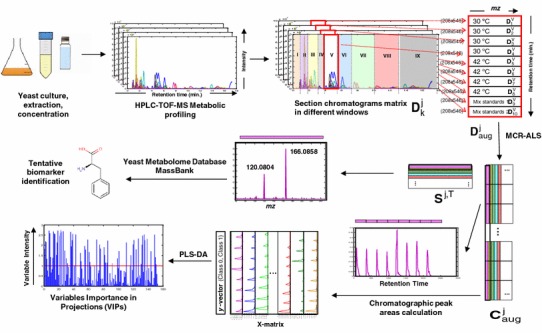
Schematic representation of the workflow following untargeted (LC–MS) data generation. The workflow involved experimental analysis, data pre-processing and data analysis in order to identify possible biomarkers (yeast metabolites)

No further arrangement was required for the single TIC data matrix. In contrast, every full scan individual preprocessed MS chromatogram data matrix was divided in ten separate submatrices corresponding to different time windows. This MS chromatogram window subdivision was done manually according to peak shape and peak density, and more specifically, not to miss those chromatographic peaks that could change with temperature. The first time window was discarded for MCR-ALS analysis since it only contained signal background and noise, i.e. all chromatograms from blanks, standards and yeast samples had the same shape profile at that initial time window. Therefore a final number of nine windows (j = I, II,…, IX) were selected from every full scan MS chromatogram data matrix of the ten analyzed samples (4 control yeast samples, k = 1,2,3,4; 4 temperature stressed yeast samples, k = 5,6,7,8 and two standard mixture samples, k = 9,10) (see Fig. 1). Therefore, for each of the ten analyzed samples, k = 1,…, 10, nine time windows were obtained, j = 1, …, 9 giving the individual data submatrices ${\mathbf{D}}_{\text{k}}^{\text{j}}$. As can be seen in Fig. 1, individual data submatrices (${\mathbf{D}}_{\text{k}}^{\text{j}}$) corresponding to the same chromatographic window for the different analyzed samples were arranged in nine column-wise augmented data matrices. The dimensions of these nine column-wise augmented data matrices depended on the dimensions of the selected time windows (selected retention times). Thus, from window I to window IX the augmented matrices dimensions were: 1010 × 546, 1110 × 546, 2210 × 546, 2910 × 546, 2080 × 546, 2760 × 546, 3410 × 546, 3210 × 546 and 4010 × 546. In every case the first dimension refers to the sum of the retention times of the ten included samples (4 control, 4 stressed samples and 2 standards), and the second dimension is equal to the number of *mz* values included in the analysis, which were in all cases 546 *mz* values (from 55 to 600 Da).

### Principal component analysis

Principal component analysis (PCA) was used for initial exploration of the behaviour of yeast samples metabolic profiles according to temperature changes. PCA compresses the information contained in the original variables into a small number of new orthogonal variables (components) built from linear combinations of the original variables explaining most of the measured data variance (Wold et al. 1987; Esbensen and Geladi 2009). Plots of firsts components are usually enough to explore the main sources of variance in the original data. Here PCA was performed on the TIC chromatograms of the eight yeast samples at the two culture temperatures (30 °C and 42 °C).

### Partial least squares-discriminant analysis (PLS-DA)

PLS-DA (Barker and Rayens 2003) is a PLS regression method (Geladi and Kowalski 1986b; Wold et al. 2001) which correlates a set of response variables **y** to a set of predictor variables **X**, where **y** is a set of binary variables of describing the categories of **X**. PLS-DA estimates in an very efficient way the best linear combinations of the independent original **X**-values (called latent variables, LV), which correlate optimally with the observed changes of the dependent variable, **y**. PLS-DA tries to build a model that maximizes the covariance between **X** and **y** with a minimum number of latent variables. For every latent variable, a vector of weight coefficients shows what **X**-variables are best combined to form the **X**-scores vector.

This method was used in this work to investigate what metabolites could be more influenced by temperature changes on yeast cultures. PLS-DA regression was applied to optimally model class variable **y** (samples cultured at 30 °C categorized in class 0 and samples cultured at 42 °C categorized in class 1, which described temperature changes in relation to the observed changes in the predictor variables (**X** matrix) (Wold 1966; Geladi and Kowalski 1986a, b; Wold et al. 2001). In this work, two PLSA-DA analysis were performed. In a first analysis, PLS-DA was applied to the **X** TIC data matrix (with dimensions of 8 × 2020), as in PCA. In a second analysis, PLS-DA was applied to the **X** TIC data matrix (with dimensions of 8 × 91), containing the peak areas of the components resolved by MCR-ALS in the full scan MS chromatographic analysis of the eight yeast samples at the two temperatures (see Sect. 3.5).

To investigate the more influent variables (peak retention times and possible metabolites associated to them) in the PLS-DA model, Variable Importance in Projection (VIP) scores (Wold et al. 1993; Wold 1995; Wold et al. 2001) were calculated. VIP scores (Wold et al. 1993) are a weighted sum of squares of PLS weights for each variable and measure the contribution of each predictor variable to the model. It is frequently used as a parameter for variable selection (Chong and Jun 2005; Rajalahti et al. 2009; Andersen and Bro 2010). For a given model and problem there is one VIP-vector, summarizing the contribution of the selected number of components on the prediction of the *y* variable (Wold et al. 2001). On the other hand, since the average of squared VIP score is equal to 1, the ‘greater than one’ rule is used as a criterion for variable selection (Chong and Jun 2005).

### Multivariate curve resolution-alternating least squares (MCR-ALS)

The goal of this analysis was to resolve the maximum number of individual elution profiles and pure mass spectral profiles of the possible metabolites extracted from the investigated yeast samples. MCR-ALS is chemometric method which allows for the resolution of multiple components in unknown unresolved mixtures from chromatographic systems, including strongly coeluted, overlapped and embedded peaks.

In the particular case under study, the MCR bilinear model is mathematically described according to:

1 $${\mathbf{D}}_{\text{k}}^{\text{j}} = {\mathbf{C}}_{\text{k}}^{\text{j}} S^{\text{j,T}} + {\mathbf{E}}_{\text{k}}^{\text{j}} \; {\text{for}}\;j = {\text{I}},\,{\text{II}}, \ldots ,\,{\text{IX}}\;{\text{windows}}\;{\text{and}}\;k = 1,\,2, \ldots ,10\;{\text{samples}}$$

Rows of data matrices ${\mathbf{D}}_{k}^{j}$ are the different elution times of the samples chromatographic analysis. Columns of data matrices ${\mathbf{D}}_{k}^{j}$ are the mass spectra recorded at the different elution times. ${\mathbf{C}}_{\text{k}}^{\text{j}}$ is the matrix of MCR-ALS resolved elution profiles in window *j* and sample *k*, and ${\mathbf{S}}^{\text{j,T}}$ is the matrix of their corresponding pure mass spectra. These resolved pure mass spectra can be then used for the identification of the different metabolites. ${\mathbf{E}}_{\text{k}}^{\text{j}}$ contains the unexplained variance related to background and noise contributions not modelled by ${\mathbf{C}}_{k}^{j}$ and ${\mathbf{S}}^{\text{j,T}}$.

This data analysis can be extended to the simultaneous analysis of the different control and stressed yeast samples and standard metabolite mixture samples (chromatographic runs), which facilitated the resolution of the coeluted metabolites simultaneously present in the yeast samples. Data submatrices ${\mathbf{D}}_{\text{k}}^{\text{j}}$ corresponding to the same time window are settled one on the top of the other (column-wise augmented matrices In this new data arrangement, the new column (*mz*) vector subspace is the same for all sample matrices. The new column-wise augmented data matrix ${\text{D}}_{\text{aug}}^{\text{j}}$ can be decomposed similarly using the bilinear model equation:

2 $${\mathbf{D}}_{\text{aug}}^{\text{j}} = \left[ \begin{gathered} {\mathbf{D}}_{1}^{\text{j}} \hfill \\ {\mathbf{D}}_{2}^{\text{j}} \hfill \\ {\mathbf{D}}_{3}^{\text{j}} \hfill \\ \vdots \hfill \\ {\mathbf{D}}_{10}^{\text{j}} \hfill \\ \end{gathered} \right] = \left[ \begin{gathered} {\mathbf{C}}_{1}^{\text{j}} \hfill \\ {\mathbf{C}}_{2}^{\text{j}} \hfill \\ {\mathbf{C}}_{3}^{\text{j}} \hfill \\ \vdots \hfill \\ {\mathbf{C}}_{10}^{\text{j}} \hfill \\ \end{gathered} \right]{\mathbf{S}}^{{{\text{j}},{\text{T}}}} + \left[ \begin{gathered} {\mathbf{\rm E}}_{1}^{\text{j}} \hfill \\ {\mathbf{E}}_{2}^{\text{j}} \hfill \\ {\mathbf{E}}_{3}^{\text{j}} \hfill \\ \vdots \hfill \\ {\mathbf{E}}_{10}^{\text{j}} \hfill \\ \end{gathered} \right] = {\mathbf{C}}_{\text{aug}}^{\text{j}} {\mathbf{S}}^{{{\text{j}},{\text{T}}}} + {\mathbf{E}}_{\text{aug}}^{\text{j}} \;{\text{for}}\;j = {\text{I}},{\text{II}}, \ldots ,{\text{IX}}\;{\text{windows}}$$

The detailed procedure followed in the MCR-ALS analysis of every window, ${\mathbf{D}}_{\text{aug}}^{\text{j}}$ is shown in Fig. 1. Nine chromatographic windows were considered for MCR-ALS analysis. These nine chromatographic windows (*j* = I, II, …, IX) covered the full investigated time range. Ten submatrices corresponding to the ten samples (*k* = 1, 2, …, 10) were considered in each augmented data matrix ${\mathbf{D}}_{\text{aug}}^{\text{j}}$ for all studied windows. Every individual data matrix (one window, one sample) had a number of rows equal to the total number of recorded elution times in the considered chromatographic region, although the total number of considered rows (retention times) did not exactly match among the different considered sample submatrices, ${\mathbf{D}}_{\text{k}}^{\text{j}}$. The number of columns was always equal to the same number of considered *mz*. ${\mathbf{C}}_{\text{aug}}^{\text{j}}$ has the resolved augmented elution profiles of the resolved peaks. ${\mathbf{S}}^{{{\text{j}},{\text{T}}}}$ is the matrix of pure mass spectra of the resolved coeluted compounds, and ${\mathbf{E}}_{\text{aug}}^{\text{j}}$ matrix is the noise and background signal absorption not explained by the model described by ${\mathbf{C}}_{\text{aug}}^{\text{j}}$ and ${\mathbf{S}}^{\text{j,T}}$(Tauler 1995).

Before starting the Alternating Least Squares (ALS) iterative process to solve Eqs. (1) and (2), the number of components is initially estimated by principal component analysis (PCA) (Wold et al. 1987) or more simply, by the singular value decomposition (SVD) (Golub and Loan 1996). In the present study, the applied constraints have been non-negativity and spectra normalization. Non-negativity was applied to chromatographic and mass spectra profiles and normalization constraint was applied to the pure mass spectra profiles to fix their scale during ALS optimization. See (Tauler and Barceló 1993; Tauler 1995; Tauler et al. 1995; de Juan et al. 2009; Tauler et al. 2009) for further details of MCR-ALS method and constraint implementation.

Figures of merit of the MCR-ALS optimization procedure are the percent lack of fit, which is the difference among the input data ${\mathbf{D}}_{\text{aug}}^{\text{j}}$ and the data reproduced from the product obtained by MCR-ALS (${\mathbf{C}}_{\text{aug}}^{\text{j}} {\mathbf{S}}^{\text{j,T}}$); and the percent of explained variance (R^2^).

Due to the high selectivity of pure component mass spectra, rotation ambiguities were practically reduced to a minimum. Only in cases where strongly coeluted peaks have common molecular ions, it is expected to have some degree of rotation ambiguity. Moreover the simultaneous analysis of multiple data matrices, including those from the analysis of the standard mixture samples, reduced more the possible presence of rotation ambiguities associated to MCR-ALS solutions of the augmented window data matrices.

Full scan LC–MS data matrices of control and temperature stressed samples, together with those of the standard mixture samples, were simultaneously analysed by MCR-ALS (see Sect. 3.2 and Eq. 2). First four matrices were the LC–MS full scan data matrices of the control yeast samples (cultured at 30 ºC). Second four matrices were the LC–MS full scan data matrices of the stressed yeast samples (cultured at 42 ºC) and last two matrices were the full scan LC–MS data matrices of the two standard mixtures. The later were included to check that MCR-ALS method was appropriately applied to separate coeluted chromatographic peaks of the components of the standard mixtures. As it was already mentioned in Sect. 3.2, since the simultaneous resolution of the whole LC–MS full scan chromatogram would give an augmented data matrix of dimensions (20200 × 546), the complete chromatogram obtained for each individual sample was sectioned in nine windows, subdividing then the MCR-ALS analysis in nine MCR-ALS differentiated analysis of the corresponding data submatrices, ${\mathbf{D}}_{\text{k}}^{\text{j}}$, *j* = I, II, …, IX (see Sect. 3.2 and Fig. 1).

## Metabolite identification

Due to the high number of peaks generated from a metabolomic analysis and in order to identify yeast metabolites, the application of MCR-ALS was used to facilitate the resolution of the coeluted and embedded chromatographic peaks. Otherwise, resulting MS chromatograms were too complex to process them at once and for the non-target searching of the individual components. Results from the ALS optimization are shown as: resolved eluted profiles, ${\mathbf{C}}_{\text{aug}}^{\text{j}}$ matrices, and pure mass spectra, ${\mathbf{S}}^{\text{j,T}}$ matrices (see Eq. 2). Peak areas of the resolved elution profiles were used to investigate possible temperature effects and mass spectra of the corresponding compounds were used for yeast metabolites identification and confirmation (Fig. 1). Since LTQ-Orbitrap instrument allowed for a very accurate mass measurement of four decimal places, possible metabolites candidates were investigated according to their accurate mass (positively ionized) value. Most abundant measurements of *mz* of all resolved pure mass spectra, ${\mathbf{S}}^{\text{j,T}}$, were used to match a metabolic feature to a single or small number of molecular formula in combination with chemical and biological knowledge. Accurate *mz* data were searched in Yeast Metabolome Database (YMDB) (Jewison et al. 2012). The candidates were checked in full scan MS chromatogram from LTQ-Orbitrap direct data acquisition having full MS accuracy. MZmine 2 framework (Pluskal et al. 2010) was used to search the resolved peaks in the LTQ-Orbitrap original raw data, taking as a reference the peak retention time obtained in the MCR-ALS eluted profiles matrix and the molecular formula matched in YMDB. Mass tolerances of 0.01 millimass units (mmu) were allowed for matching a particular molecular formula when searched in LTQ-Orbitrap full scan MS chromatograms. In this way, it was ensured that considered MCR-ALS resolved elution profiles matched with the ones originally present in raw MS chromatograms. Further confirmation of the MCR-ALS resolved candidates (metabolites) was done by comparison with the metabolite mass spectrum from the MassBank mass public spectral database (Horai et al. 2010).

Although some ion types were expected (protonated peaks, isotope peaks) others were not expected (adduct ions). Initial identifications were refined using previous analyses of the yeast metabolome (Canelas et al. 2009; Beltran et al. 2012). Predicted exact mass for different adducts were calculated using the Mass Spectrometry Adduct Calculator from Metabolomics Fiehn Lab webpage (Huang et al. 1999). Identified metabolites were further functionally and metabolically characterized using the KEGG database (Kanehisa et al. 2012). Section 5.4 gives an example of detailed application of the procedure described above.

## Results and discussion

### PCA and PLS-DA of MS-TIC chromatograms

**Fig. 2:**
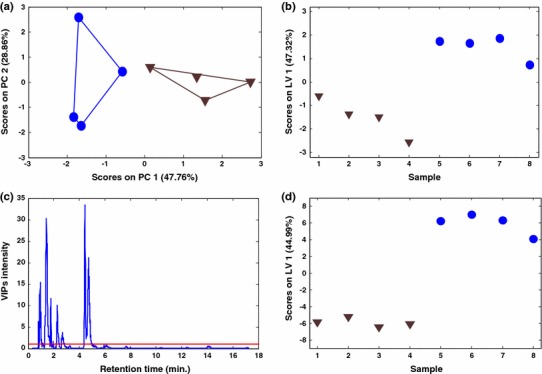
**a** PCA scores plot for the eight yeast samples (MS TIC chromatograms). Convex hulls are drown around each yeast culture temperature group with the *same color* as the *corresponding symbols*. **b** PLS-DA scores plot for the eight yeast samples at the two temperatures using their MS TIC chromatograms. **c** Variables importance in projection (VIP) plot resulting from PLS-DA analysis of full scan MS TIC yeast chromatograms. *Horizontal red line* shows the threshold value used to select the variables with the most important VIP scores. **d** PLS-DA scores of autoscaled chromatographic peak areas obtained by MCR-ALS analysis of full scan MS chromatographic data of the analysed yeast samples. In **a**, **b** and **d** *blue solid circles* are control samples cultured at 30 °C and *brown triangles* are yeast samples cultured at 42 °C (Color figure online)

When principal component analysis (PCA) was applied to the mean-centered MS TIC data matrix (with 8 samples and 2020 measured chromatographic retention times), three principal components already explained 88.83 % of data variance. In Fig. 2a, scores of the first two components are given. PC1 explains 47.76 % of the data variance and separates the samples in relation to the yeast culture temperatures. Samples grouped in the negative side of PC1 axis were grown at 42 ºC and samples grouped on the positive side of PC1 axis are the control samples grown at 30 ºC. Variances explained by PC2 and PC3 are related to other unknown variability sources not dependent of temperature.

Partial least-squares discriminant analysis (PLS-DA) was applied to investigate the more relevant variables (peak retention times) related to the discrimination between control and stressed yeast samples. The performance of PLS-DA model was also calculated on the mean-centred MS TIC data matrix. PLS-DA was assessed by using leave-one-out cross-validation method (adequate for a small number of samples as in this study). ***y*** vector containing the class labels was also mean-centred. First PLS latent variable (LV1) already accounted for 47.32 % of **X** data variance and for 86.66 % of the dependent variable ***y*** (low temperature control samples and high temperature stressed yeast samples). This confirms again that the main source of variance in TIC chromatograms was related to temperature changes. In the scores plot of PLS-DA (see Fig. 2b), the two groups of samples (control and temperature stressed) were clearly distinguished. To help the visualization of the more influent variables PLS-DA VIP values were calculated (see Sect. 3.3 and Fig. 2c).

Due to the strong co-elution among multiple chromatographic peaks at the same retention times in all TIC chromatograms, the evaluation of the relative importance of the different variables (peak retention times) on temperature changes was rather difficult and further analysis was performed using MCR-ALS analysis of full scan MS chromatograms. This allowed the improved mathematical resolution of the coeluted peak profiles and the estimation of their corresponding pure mass spectral profiles, and their further identification (see below).

### MCR-ALS of full scan LC–MS chromatograms

A total number of nine column-wise augmented data matrices, each one corresponding to one of the nine windows, were analysed separately by MCR-ALS. All MCR-ALS explained variance (R^2^) percentages were higher than 98 %. A relatively large number of MCR-ALS components (resolved peaks) were needed to explain properly the observed data variance and patterns. Not all MCR-ALS resolved components corresponded to true chromatographic peaks which could be assigned to separate metabolites, since other possible signal contributions such as the background and solvent contributions could also be present.

**Fig. 3:**
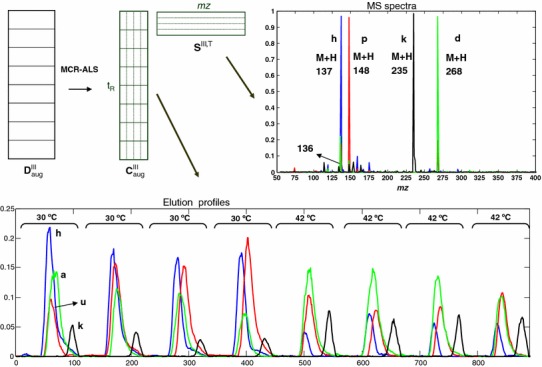
Example of MCR-ALS resolution (Multivariate curve resolution-alternating least squares) simultaneously applied to the column-wise augmented data matrix (${\mathbf{D}}_{\text{aug}}^{\text{III}}$) corresponding to time window III including the control and the stressed yeast samples. ${\mathbf{C}}_{\text{aug}}^{\text{III}}$ is the matrix of MCR-ALS resolved elution profiles. ${\mathbf{S}}^{\text{III,T}}$ is the matrix of MCR-ALS resolved MS pure spectra. Compound labels identification in this example are: *h* hypoxanthine, *p* palmitic acid, *r* arbitol/ribitol, *d* deoxyguanosine

Figure 3 is an example of MCR-ALS applied to the column-wise augmented data matrix (${\mathbf{D}}_{\text{aug}}^{\text{j}}$) corresponding to time window III (elution times from 1.82 to 2.69). In this example, the two standard mixture samples were omitted from the figure because there was no standard compound eluting in this time window. As it can be seen in Fig. 3, the four coeluted components (h, p, r, d) were successfully separated by MCR-ALS analysis. Elution profiles (${\mathbf{C}}_{\text{aug}}^{\text{III}}$) and pure mass spectra (${\mathbf{S}}^{{{\text{III}},{\text{T}}}}$) are shown. The four contributions (h, p, r, d) were identified by their mass spectra as explained in Sects. 4 and 5.4 for their further metabolite identification). There were other components (not shown) which were sections of chromatographic peaks corresponding to windows II and IV. There were also some minor noise interferences without chromatographic peak shape and very imprecise spectra, which were finally not shown in the figure for clarity.

As stated before, when resolving the column-wise augmented data matrices of every window j, ${\mathbf{D}}_{\text{aug}}^{\text{j}}$ with MCR-ALS, *mz* resolution was restricted to one integer mass value to facilitate its numerical analysis (see Sect. 3.1). This resolution was generally enough to resolve the elution profiles of the coeluted metabolites, and allowed the simultaneous estimation of their full scan MS spectra at this limited resolution. In some cases, however, this was not sufficient, and an optimal resolution could not be achieved. For instance, when two peaks were overlapped in the same elution profile but giving the same MS, after checking their exact mass in the high resolution raw data these two compounds resulted to have slightly different masses. Therefore, to allow the separation of these two chromatographic peaks as different components higher resolution data should be processed by MCR-ALS.

Once the whole set of column-wise augmented data matrices corresponding to the nine time windows were analysed by MCR-ALS, the areas of all MCR-ALS resolved peaks for each component profile were calculated and arranged in a data table. In total, 91 components were resolved and their peak areas calculated for every one of the eight analysed yeast samples, i.e. a table with a total number of 8 × 91 peak areas was obtained.

Metabolite profiles in all window profiles were strongly overlapped and their proper resolution was strongly facilitated by the proposed MCR-ALS analysis. It might be argued that similar results could have been achieved using LC–MS in single ion monitoring (SIM) mode once their characteristic molecular MS ions were identified (target analysis). However this analysis would have required the exploration of such a large number of possibilities that it would have made this metabolomic study very tedious and impractical, if not unaccurate in many cases. Moreover, for the goals of the present study, the larger the number of possible unknown metabolites (non-target analysis) simultaneously analyzed and resolved the more interesting could be the conclusions derived from the obtained results.

### PLS-DA of chromatographic peak areas

PLS-DA was applied to the peak areas of all 91 MCR-ALS resolved elution profiles, for control and treated yeast samples (X matrix with dimensions of 8 × 91). Prior to PLS-DA model calculation, the peak areas were autoscaled to give equal relevance to their possible change due to the temperature differences in control and treated yeast samples. PLS-DA model was developed to investigate what metabolite peak areas were more important in the discrimination between yeast culture samples at 30 and 42 ºC and to identify potential metabolites markers of the temperature effects on yeast metabolism. No outlier samples were detected using a leave-one-out cross-validation (adequate strategy for a small number of samples). One PLS-DA component was enough to explain most of the class variance (98.02 % variance) using the 44.99 % of **X** variance related to the changes on chromatographic peak areas of the resolved components, with specificity and sensitivity values equal to 1 for each class.

In Fig. 2d, the projection of the first latent variable (LV1) scores is given; two groups of samples are clearly distinguished. Yeast samples at 30 ºC were projected on the negative scores axis whereas yeast samples at 42 ºC were projected on the positive scores axis.

**Fig. 4:**
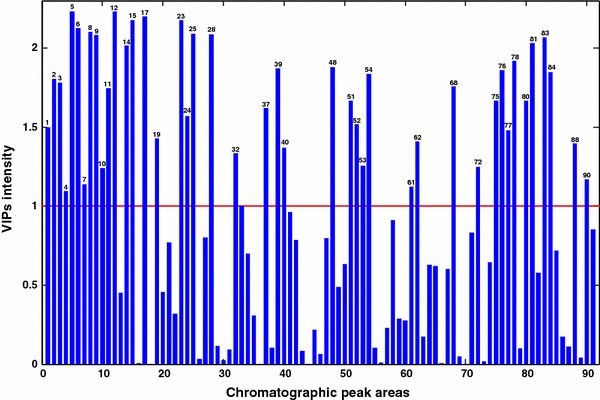
Variables importance in projection (VIP scores) plot resulting from PLS-DA analysis of the autoscaled chromatographic peak areas obtained by MCR-ALS analysis of yeast samples. *Horizontal red line* shows the threshold value selecting the most important variables (Color figure online)

VIP values (Eriksson et al. 2006) calculated from PLS-DA model (see Sect. 3.3) revealed what variables (metabolites) were more important to discriminate the effects of temperature changes on yeast metabolism. Variables whose VIP values were higher than 1.0 were considered as potential indicators (Chong and Jun 2005) of these effects (see Fig. 4). Results are listed in Table 2. PLS-DA VIPs (Fig. 4) and the corresponding PLS-DA weights provided a summary of PLS-DA results. In this table chromatographic peaks increasing (‘Ups’) or decreasing (‘Downs’) at 42 ºC relative to the control samples cultured at 30 ºC are given.

### Tentative identification of possible biomarker compounds

Identification of the metabolites corresponding to all 91 resolved chromatographic peaks was attempted. Taking as example the pure MS spectrum of one of the components resolved by MCR-ALS (at window IV, component 12, peak 40 on Table 2), its corresponding metabolite identification was performed in the following way: Its main integer mass value was 166 (molecular mass positively ionized). When this mass value was searched in the YMDB database, possible candidates were l-methionine (R)-S-oxide, D/l-Phenylalanine, 4-Pyridoxolactone, *N*-Formylanthranilic acid and 7-Methylguanine. These were the yeast metabolites that when positively ionized, [M + H]^+^, give the value of 166 as the most abundant *mz* of its mass spectrum. When these candidates were searched in the raw full scan MS chromatograms, using then high resolution *mz* values through MZmine2 framework (Pluskal et al. 2010), D/l-Phenylalanine was the selected candidate that better matched with the resolved chromatographic peak. Its accurate mass was 166.0858, which when searched in MassBank database (Horai et al. 2010) was confirmed to be the spectrum of d/l-Phenylalanine (with an accurate mass of 166.0868). Some MS pure spectra resolved by MCR-ALS gave additional less intense signal product ions which could be also used for identification and confirmation of possible metabolite candidates. For instance, in the case of the MCR-ALS resolved pure MS spectrum of d/l-Phenylalanine another ion mass was detected at 120.0804, which matched very well with a product ion of metabolite d/l-Phenylalanine at 120.0813 in the MassBank data base. This should be considered a clear additional advantage of the proposed MCR-ALS strategy which is difficult to be achieved using traditional direct identification approaches. MCR-ALS resolution strategy allows the simultaneous identification of both molecular and product ions in the spectrum of every resolved component.

Following the same procedure, a total number of 65 metabolites were tentatively identified out of the 91 potentially different chromatographic peaks (Table 2). In most cases (54 out of 65), observed *mz* values differed from the calculated ones by less than 50 ppm (Table 2), and this error was considered acceptable given the methodology used in this work. All metabolites displayed in Table 2 are either bona fide yeast metabolites classified as such in the YMBD (see YMDB numbers in Table 2) or they have been identified in similar yeast metabolome studies (Canelas et al. 2009; Beltran et al. 2012). Identified metabolites include 17 out of the 20 protein amino acids, plus l-Ornithine, although l-Serine and l-Glutamine. Other major components of the yeast metabolome were identified, as nucleosides and their derivatives, vitamins, glycerol and some organic acids and lipids (Table 2). In summary, the proposed methodology correctly identified many of the major components of the yeast metabolome. Further work and improvement of the proposed methodology is persued to allow the complete description of the more important metabolites present in yeast cells.

### Biological interpretation of the changing metabolites

Results obtained in previous Sects. 5.3 and 5.4 showed that some of the MCR-ALS resolved compounds displayed a relative increase or decrease of their abundances (relative chromatographic areas) depending on the culture temperature (42 vs. 30 ºC). Tentative identification of metabolites whose abundance increased (Ups) or decreased (Downs) at 42 ºC relative to the standard 30 ºC culture temperature is displayed in Table 1. In this case, almost 80 % of the peaks showing a significant change of their area due to temperature were tentatively identified. Functional analyses of the altered metabolites did not allow an accurate assessment of the metabolic pathway changes underlying the acclimatisation of the yeast cell to growth at high temperatures. However, it is noticeable the increase of two pentose alcohol, tentatively identified as ribitol and arabitol (Tables 1, 2), coupled to the decrease of glycerol. Yeast is known to maintain their osmotic balance by modifying their internal concentration of glycerol and/or sugar alcohols (Hohmann 2002). Although *S.* *cerevisiae* is believed to only use glycerol for this protective function, the observed changes may reflect a re-equilibrium in osmolite concentrations as a response to the high growth temperature. Similarly, decrease of two structural lipids/organic acids (palmitic acid and the lysophospholipid tentatively identified as LysoPC(18:1(11Z)) may reflect an alteration on the yeast lipid composition to adapt membrane fluidity to the high temperatures. Finally, it is known that continuous growth at high temperatures induces a switch in yeast metabolism towards respiration from fermentation (Mensonides et al. 2013)). Therefore, it is conceivable that some of the observed changes, like the increase of short/medium-length organic acids adipic, caprylic, and capric acids, or the decrease of fermentation sub-products, like acetate and glycerol, may reflect this metabolic adaptation to temperature changes. Further interpretation of the observed changes will require a more accurate investigation and knowledge of biochemical pathways of yeast growing under different temperatures.

**Table 1:** Temptative identification of yeast metabolites associated to chromatographic peaks changing their areas when culture temperature changed from 30 to 42 °C

Ups				Downs			
Peak number	C-number	Metabolite	Weight	Peak number	C-number	Metabolite	Weight
3	C06104	Adipic acid	0.1401	1	C00033	Acetic acid	−0.1285
5	C05853	2-Phenylethanol	0.1568	2	C00097	l-Cysteine	−0.141
6	C00077	l-Ornithine	0.1532	4			−0.1098
11	C00864	Pantothenate	0.1388	7	C00116	Glycerol	−0.1121
12	C01571	Capric acid	0.1569	8	C00147	Adenine	−0.1523
14	C00474	Arabitol/ribitol	0.1491	9	C00262	Hypoxanthine	−0.1516
15	C00559	Deoxyadenosine	0.1549	10	C00249	Palmitic acid	−0.1169
17	C06423	Caprylic acid	0.1558	23	C00791	Creatinine	−0.155
19	C00120	Biotin	0.1255	24			−0.1315
25	C00474	Arabitol/ribitol	0.1518	32		LysoPC(18:1(11Z))	−0.1213
28	C01087	2-Hydroxyglutaric acid	0.1517	37	C00902	2-Oxohexanoic acid	−0.1337
40	C00079	l-Phenylalanine	0.1229	39	C00160	Glycolic acid	−0.1437
51			0.1356	48	C00250	Pyridoxal	−0.1439
54	C00049	l-Aspartic acid	0.1423	52	C02794	L-3-Hydroxykynurenine	−0.1293
62	C00041	l-Alanine	0.1247	53	C00082	l-Tyrosine	−0.1176
72			0.1173	61	C00152	l-Asparagine	−0.1112
75	C00114	Choline	0.1356	68			−0.1392
76	C07113	Acetophenone	0.1432	77	C02059	Phylloquinone	−0.1277
80			0.1356	78	C00378	Thiamine	−0.1454
81			0.1496	84	C00192	Hydroxylamine	−0.1428
83			0.151	88	C01346	dUDP	−0.1242
90			0.1135

**Table 2:** Tentative adscription of yeast metabolites to observed mass values for the different chromatographic peaks (not only those showing area changes when culture temperature change)

Peak number	Retention time	Highest mass ion	Proposed metabolite	KEGG C-number	YMDB	Adduct	Adduct m/z	error mz (ppm)	>50 ppm
1	1.38	102.0547	Acetic acid	C00033	YMDB00056	M + ACN + H	102.0549	1.96	
2	1.33/1.69	123.0552	l-Cysteine	C00097	YMDB00046	M + 3ACN + 2H	123.0569	13.81	
3	1.7–2	293.1167	Adipic acid	C06104		2M + H	293.1231	21.83	
5	1.8	267.1452	2-Phenylethanol		YMDB01072	2M + Na	267.1355	36.13	
6	1.48	155.0811	l-Ornithine	C00077	YMDB00353	M + Na	155.0791	12.90	
7	1.9–2	156.0656	Glycerol	C00116	YMDB00283	M + ACN + Na	156.0631	16.02	
8	2.1	136.0619	Adenine	C00147	YMDB00887	M + H	136.0618	0.73	
9	2.26	137.0457	Hypoxanthine		YMDB00555	M + H	137.0458	0.63	
10	2.28–2.38	148.0965	Palmitic acid	C00249	YMDB00069	M + H + K	148.1053	59.42	*
11	1.85–2.5	220.1175	Pantothenate	C00864	YMDB00203	M + H	220.1180	2.16	
12	2.1	211.1119	Caproic acid	C01571	YMDB00677	M + K	211.1095	11.37	
13	2.3	268.1028	Deoxyguanosine	C00330	YMDB00505	M + H	268.1041	4.76	
14	2.59	235.1192	Arabitol/ribitol	C00474	YMDB00591	M + 2ACN + H	235.1288	40.83	
15	1.95	252.1088	Deoxyadenosine	C00559	YMDB00503	M + H	252.1091	1.09	
17	2.1	167.0922	Caprylic acid	C06423	YMDB00676	M + Na	167.1042	71.81	*
19	1.8	245.0949	Biotin	C00120	YMDB00282	M + H	245.0955	2.35	
20	3.6	209.1032	Thymine	C00178	YMDB00885	M + 2ACN + H	209.1033	0.48	
21	4.1	195.0871	Uracil	C00106	YMDB00098	M + 2ACN + H	195.0877	3.08	
23	2.7	114.0659	Creatinine	C00791		M + H	114.0662	2.63	
25	2.6	235.1192	Arabitol/ribitol	C00532	YMDB00591	M + 2ACN + H	235.1288	40.83	
26	3.1–3.3	494.3212	LysoPC(16:1(9Z))		YMDB02210	M + H	494.3241	5.90	
28	2.7	166.072	2-Hydroxyglutaric acid	C01087	YMDB00059	M + NH4	166.0710	6.02	
30	3.3–3.4	192.1588	l-Arginine	C00062	YMDB00592	M + NH4	192.1455	69.22	*
31	2.7	184.0633	L-2-Aminoadipate		YMDB00999	M + NA	184.0580	28.65	
32	2.85	522.3528	LysoPC(18:1(11Z))		YMDB02211	M + H	522.3554	5.01	
34	4.3	162.0576	Cystine	C01420	YMDB00861	M + 2ACN + 2H	162.0457	73.29	*
35	5.9	150.0583	l-Methionine	C00073	YMDB00318	M + H	150.0583	0.00	
37	2.3	148.0965	2-Oxohexanoic acid	C00902	YMDB00388	M + NH4	148.0968	2.03	
38	1.8–2	205.0673	2-Oxobutanoate	C00109	YMDB00071	2 M + H	205.0707	16.58	
39	4.5–4.6	159.0761	Glycolic acid	C00160	YMDB00807	M + 2ACN + H	159.0764	1.89	
40	4.5–4.6	166.0858	l-Phenylalanine	C00079	YMDB00304	M + H	166.0863	3.01	
41	4.7–4.8	205.0972	l-Tryptophan	C00078	YMDB00126	M + H	205.0972	0.12	
48	5–5.3	209.0917	Pyridoxal	C00250	YMDB00392	M + ACN + H	209.0920	1.43	
49		132.102	l-Isoleucine	C00407	YMDB00038	M + H	132.1019	0.76	
49		132.102	l-Leucine	C00123	YMDB00387	M + H	132.1019	0.76	
52	5.9–6	225.0858	L-3-Hydroxykynurenine		YMDB00105	M + H	225.0870	5.26	
53	6.1–6.2	182.0804	l-Tyrosine	C00082	YMDB00364	M + H	182.0812	4.39	
54	7.1	175.0866	l-Aspartic acid	C00049	YMDB00896	M + ACN + H	175.0713	87.39	*
56		116.0705	l-Proline	C00148	YMDB00378	M + H	116.0706	0.86	
57	6.2	118.0863	Histamine	C00388	YMDB01556	M + 3ACN + 2H	118.0869	5.08	
58	6	118.0858	l-Valine	C00183	YMDB00152	M + H	118.0863	4.23	
59	6	72.0805	2-Nonanone		YMDB01383	M + 2H	72.0752	74.11	*
60	6.6–6.7	148.0605	l-Glutamate	C00025	YMDB00271	M + H	148.0605	0.00	
61	7.6–7.7	150.0778	l-Asparagine	C00152	YMDB00226	M + NH_4_	150.0873	63.30	*
62	7.6	90.0546	l-Alanine	C00041	YMDB00154	M + H	90.0550	4.44	
64	9.1	147.0764	l-Glutamine	C00064	YMDB00002	M + H	147.0764	0.00	
64	9.1	147.0764	l-Serine	C00065	YMDB00112	M + ACN + H	147.0764	0.00	
65	7	218.1384	Ergosterol	C01694	YMDB00543	M + H + K	218.1548	75.25	*
67	8.8	120.065	l-Threonine	C00188	YMDB00214	M + H	120.0655	4.16	
69	11–11.2	337.1677	Pyridoxamine	C00534	YMDB00889	2M + H	337.1871	57.53	*
70	9.5	162.1122	Octadecanoic acid	C01530	YMDB00682	M + H + K	162.1210	54.08	*
71	10.2	258.1088	Glycerophosphocholine		YMDB00309	M + H	258.1101	5.04	
73	9.7–9.8	246.0982	Deoxyuridine	C00526	YMDB00508	M + NH4	246.1084	41.54	
74	11.3–11.4	401.1649	Cortisol	C00735		M + K	401.1725	18.84	
75	11.7–11.8	146.1175	Choline	C00114	YMDB00227	M + ACN + H	146.1413	162.86	*
76	9.5	121.0648	Acetophenone	C07113	YMDB01629	M + H	121.0648	0.00	
77	10–11.3	151.123	Phylloquinone	C02059	YMDB01526	M + 3H	151.1239	5.80	
78	12.7–12.9	154.0972	Thiamine	C00378	YMDB00220	M + ACN + 2H	154.0767	133.05	*
82	12–12.1	130.0974	Methyl-3-ethyl-butanoate		YMDB01749	M + H	130.0988	11.00	
84	13.8–14.2	116.0819	Hydroxylamine	C00192		M + 2ACN + H	116.0818	0.86	
86	14.9–15	309.1377	Adenosine	C00212	YMDB00058	M + ACN + H	309.1306	22.89	
87	15.5–15.6	131.118	lignoceric acid	C08320	YMDB00684	M + 2H + Na	131.1231	38.89	
88	14.5–15	145.1079	dUDP	C01346	YMDB00746	M + H + 2Na	145.1020	40.96	
89	14.2–14.3	181.0968	Limonene	C06078	YMDB01727	M + 2Na–H	181.0964	2.21	
91	13.3	137.1069	2-Methyl-5-propylpyrazine		YMDB01504	M + H	137.1073	3.10	

## Concluding remarks

Direct analysis of preprocessed TIC chromatograms by PLS-DA resulted to be a rather limited strategy to uncover yeast growth metabolite concentration changes under different temperatures. This limited strategy did not allow for the identification of the most important metabolites related to yeast culture growth under different temperatures, due to the strong overlapping of the chromatographic peaks associated for the large number of different coeluted metabolites. The novel MCR-ALS strategy presented here allowed the resolution of coeluted chromatographic peaks, the calculation of their corresponding peak areas and the resolution of their corresponding pure MS spectra.

In this work, a new workflow for metabolite identification in untargeted metabolomics is demonstrated. The resolved pure MS spectra together with the high mass accuracy offered by the LTQ-Orbitrap enabled the identification of the resolved chromatographic peaks. A total number of 65 metabolites out of the 91 total detected were successfully identified. Changes in MCR-ALS chromatographic peak areas of some of the metabolites in control and stressed yeast samples were used to detect possible variations of metabolite concentrations at the two different culture temperatures by means of PLS-DA-VIP scores analysis. Results revealed that the concentrations of 43 metabolites were significantly changed according to the yeast culture temperature (stressing factor). Further research is proposed to complete the biochemical interpretation of the effects of temperature on yeast metabolome and confirm possible biomarkers of these effects. Preliminary analysis, indicate that some metabolites linked to cell growth were affected by temperature with a consistent pattern of temperature-driven metabolic adaptation, although changes observed are still giving an incomplete description of the temperature effects on yeast growing process. The proposed strategy can simplify considerably the biochemical LC–MS data interpretation and allow the uncovering of new targets for discovery (biomarkers).
